# Anticipating the direction of symptom progression using critical slowing down: a proof-of-concept study

**DOI:** 10.1186/s12888-022-03686-9

**Published:** 2022-01-21

**Authors:** Marieke J. Schreuder, Johanna T. W. Wigman, Robin N. Groen, Els Weinans, Marieke Wichers, Catharina A. Hartman

**Affiliations:** 1 Department of Psychiatry, Interdisciplinary Center Psychopathology and Emotion regulation (ICPE), University of Groningen, University Medical Center Groningen, Internal Postal Code: CC72, Triade Building Entrance 24, Hanzeplein 1, Groningen, 9713 GZ The Netherlands; 2 Department of Industrial Engineering and Innovation Sciences, Eindhoven University of Technology, Eindhoven, The Netherlands

**Keywords:** Transitions in psychopathology, Critical slowing down, Daily diary study, Complex systems

## Abstract

**Background:**

As complex dynamic systems approach a transition, their dynamics change. This process, called critical slowing down (CSD), may precede transitions in psychopathology as well. This study investigated whether CSD may also indicate the *direction* of future symptom transitions, *i.e.,* whether they involve an increase or decrease in symptoms.

**Methods:**

In study 1, a patient with a history of major depression monitored their mental states ten times a day for almost eight months. Study 2 used data from the TRAILS TRANS-ID study, where 122 young adults at increased risk of psychopathology (mean age 23.64±0.67 years, 56.6% males) monitored their mental states daily for six consecutive months. Symptom transitions were inferred from semi-structured diagnostic interviews. In both studies, CSD direction was estimated using moving-window principal component analyses.

**Results:**

In study 1, CSD was directed towards an increase in negative mental states. In study 2, the CSD direction matched the direction of symptom shifts in 34 individuals. The accuracy of the indicator was higher in subsets of individuals with larger absolute symptom transitions. The indicator’s accuracy exceeded chance levels in sensitivity analyses (accuracy 22.92% vs. 11.76%, z=-2.04, *P*=.02) but not in main analyses (accuracy 27.87% vs. 20.63%, z=-1.32, *P*=.09).

**Conclusions:**

The CSD direction may predict whether upcoming symptom transitions involve remission or worsening. However, this may only hold for specific individuals, namely those with large symptom transitions. Future research is needed to replicate these findings and to delineate for whom CSD reliably forecasts the direction of impending symptom transitions.

**Supplementary Information:**

The online version contains supplementary material available at 10.1186/s12888-022-03686-9.

## Background

About 86% of individuals will meet the criteria for a mental disorder at some point in their lives [1]. Given the considerable burden associated with mental disorders, there has been great interest in prevention and early intervention [2]. Successful prevention requires a solid understanding of what it means to be 'at risk' for developing psychopathological symptoms. Despite a large number of well-known characteristics that predispose individuals to psychopathology, predicting at the individual level who will or will not develop a disorder remains largely an open question. Most individuals who are considered at risk do *not* develop a disorder [3, 4]. In order to better identify those at-risk individuals who could benefit from preventive interventions, an improved prediction of the prognosis of at-risk individuals is necessary.

One route towards improved prediction is to gain more detailed knowledge on within-individual changes occurring on the verge of disorder onset. It has been suggested that the structure of symptoms, *i.e.,* the extent to which symptoms reflect a single construct and how they covary, changes as individuals improve or worsen in terms of psychopathology [5–7]. This could mean that changes in the structure of symptoms may predict future progression of symptoms. Within-individual support for this idea is currently limited to a single case study, which showed that a relapse in depression was preceded by rising covariance between symptoms [8]. Between-individual or group-level support, provided by studies that compared symptom covariance of one group (*e.g.,* individuals prior to treatment) to another group (*e.g.,* the same individuals after treatment), is abundant but warrants cautious interpretation. This has two reasons: first, these comparisons may be subject to Berkson’s bias [9], and second, between-individual findings do not necessarily translate to the within-individual level [10]. With these considerations in mind, it is noteworthy that both worsening and remitting psychopathology have been related to increased symptom covariances. Specifically, symptom covariance may be higher in individuals with persisting [11, 12] or worsening [13] symptoms compared to individuals with remitting symptoms over time, although not all studies confirmed this [14–16]. If this also holds within individuals, it could mean that high symptom covariances predispose individuals to psychopathology. However, symptom covariance has also been found to be *lower* in individuals before compared to after treatment [17–24]. This could mean that high symptom covariances are linked to mental health. For instance, symptom remission may coincide with an altered appraisal of symptoms, meaning that individuals may increasingly perceive their symptoms as belonging to a unified latent construct (*i.e.,* a disorder) [18, 20, 25]. Alternatively, the association between symptom covariance and remission could be due to the simultaneous absence of symptoms (*i.e.,* floor effects). Regardless of the inferences drawn, there seems to be an apparent paradox: increased symptom covariances might relate to both symptom worsening and remission. It is at present unclear how to reconcile this. Further, it remains largely unknown whether changing symptom covariances over time can *prospectively predict* symptom remission or worsening within individuals.

A complex dynamic systems approach to psychopathology provides a framework that unites earlier findings and can be used to address the unresolved questions described above. In complex dynamic systems, transitions are often preceded by a period during which the stability within the system gradually declines, a phenomenon known as *critical slowing down*. Otherwise unpredictable transitions – such as the extinction of a species, sudden climate changes, or a sudden transition in mental health – might thus be anticipated by monitoring a the instability of a system [26–28]. Critical slowing down has been shown to precede not only ecosystem and climate transitions [29], but also transitions between depressed and manic episodes in bipolar disorder [30] as well as relapse and remission of depression [8, 31, 32]. This means that critical slowing down, which can be assessed in repeated assessments of mental states or symptoms of psychopathology, may foresee upcoming mental health problems. Recently, the potential of critical slowing down as a warning sign for impending transitions has been extended by noting that critical slowing down has a *direction*, meaning that it involves only a specific combination of variables inthe system [33–36]. This, in turn, means that critical slowing down could expose whether a transition is directed towards, for instance, extinction of one species or the other [33]. In the context of psychopathology, exposing the direction of critical slowing down may allow for inferring whether an upcoming symptom transition is directed towards worsening or remitting symptoms. The direction of critical slowing down can be monitored using metrics similar to those described in earlier studies, namely symptom covariances (or, more specifically: the eigenvalues of the covariance matrix [33, 34, 36, 37]). Hence, the hypothesis that follows from a complex dynamic systems approach can be considered an extension of what was reported earlier, namely: a gradual alteration in the structure of psychopathological symptoms prospectively predicts whether a specific individual will experience a symptom transition towards remission (decrease of symptom severity) or worsening (increase of symptom severity).

The current study aimed to test the hypothesis that symptom changes within individuals – involving either an increase or decrease in symptoms over time – can be predicted based on the direction of critical slowing down [33]. Given that the application of complex dynamic systems principles to psychopathology is still in its infancy, we will approach our aim in two steps. First, we will provide a proof of concept by testing our hypothesis in a dataset in which principles from complex dynamic systems have already been confirmed [8, 38, 39]. These data are time series of a single individual with a history of depression who experienced a relapse (*i.e.,* a sudden increase in symptoms), and contain multiple momentary ratings of mental states per day over a period of almost eight months. We will extend earlier findings [8], which showed that critical slowing down preceded the relapse in depression, by investigating whether critical slowing down is indeed directed towards symptom worsening (as opposed to remission). Given that critical slowing down has been hypothesized to reflect a generic phenomenon, we will next investigate whether the directionality of critical slowing down generalizes to symptom transitions with varying directions and magnitudes. To this end, we will repeat the analyses in a larger dataset, which consists of 134 young adults at increased risk for mental health problems who provided daily ratings on their mental states over a period of six months [40]. We hypothesize that in the first dataset, the direction of critical slowing down [33, 34] points towards a relapse of symptoms. Similarly, in the second dataset, we hypothesize that the direction of critical slowing down corresponds to the change in symptoms reported by individuals (*e.g.,* strong critical slowing down towards improvement in individuals who experienced a large reduction of symptoms, and vice versa).

## Methods

### Study 1

The data analyzed in study 1 were extensively described elsewhere and are publicly available [39]. Briefly, this study concerned a male participant diagnosed with a history of major depressive disorder who had been using antidepressant medication for 8.5 years and wanted to taper this medication. To gain more insight into his vulnerability to depressive symptoms during this tapering period, the participant monitored his mood 10 times a day for almost eight consecutive months. During this period, the participant experienced a relapse in depressive symptoms [8]. The participant gave his consent to collect and (re)use his data [39].

### Experience sampling procedure

Experience sampling involved completing 10 questionnaires per day for a period of 239 days (almost 8 months), resulting in 1478 completed observations. Each questionnaire consisted of 50 items, of which 12 pertained to mood states. Mood-related items with negative valence (*e.g.,* feeling stressed) were rated on a 7-point Likert scale ranging from -3 to 3, while items with positive valence (e.g., feeling content) were rated on a scale that ranged from 0 to 7. We rescaled items with negative valence to maintain a consistent interpretation. The daily assessments were complemented with weekly assessments of the depression subscale of the Symptom Checklist Revised [41]. The latter were used to monitor changes in severity of symptoms, as an indicator of a relapse in depression.

### Study 2

Data analyzed in Study 2 were retrieved from the TRAILS TRANS-ID study, which has been described in detail elsewhere [40]. TRAILS TRANS-ID included 134 participants from an ongoing prospective cohort study, named Tracking Adolescents’ Individual Lives Survey (TRAILS). TRAILS was designed to monitor mental health from childhood to adulthood through bi- or tri-annual assessments, and includes a general population and a clinical cohort [42]. Participants were eligible to join the clinical cohort (TRAILS CC) if they (i) were between 10 and 12 years old and (ii) had been referred to a child psychiatric outpatient clinic in the Northern Netherlands earlier in life. Given the latter criterion, TRAILS CC participants were considered at increased risk for psychopathology. This was confirmed by previous descriptive studies, which showed that TRAILS CC participants had more mental health problems than individuals from the general population [42, 43]. Of the 1264 children who were eligible for TRAILS CC, 543 (43.0%) agreed to participate. These responders did not differ from non-responders (*N*=721, 57.0% of the eligible children) in terms of age, sex, parental educational attainment, age at referral, or severity of psychopathology [42]. After their inclusion in TRAILS CC, participants were invited for multiple follow-up assessment waves. Prior to each of these waves, informed consent was obtained from parents and/or participants. The TRAILS study was approved by the Dutch Central Committee on Research Involving Human Subjects (CCMO) and in accordance with the ethical standards laid down in the 1964 Declaration of Helsinki as revised in 2008.

At the age of 23 years old, TRAILS CC participants who were still participating in the study and had given their consent to be approached for future assessments (*N* = 443) were invited to a six-month daily diary study (TRAILS TRANS-ID). In total, 142 individuals responded to this invitation and 134 individuals (30.2% of the total sample) were eventually included in the study. In a manuscript that is currently in progress, we found that these 134 participants were similar to TRAILS CC participants (*N*=309) who did not decide to participate in TRAILS TRANS-ID in terms of demographic, social, economic, psychological, and physical characteristics. For analyses, we included those individuals who completed a diagnostic interview both prior to and immediately after the diary study (*N*=122, 91.0% of those who commenced the study). Twelve individuals (9.0%) who did not complete the diagnostic interviews after the diary study were thus excluded from analyses. Excluded individuals did not differ from those who were included in terms of age, sex, socio-economic status, and diagnostic status at baseline [40]. TRAILS TRANS-ID was approved by the local Medical Ethical Committee (reference no. 2017/203). All participants provided written informed consent.

### Diary procedure

For a period of six consecutive months, participants completed a questionnaire (diary) every evening concerning the past day, resulting in a maximum of 183 observations per participant. Participants received these questionnaires through a link sent in a text message to their mobile phones. Each questionnaire consisted of 58 items pertaining to positive mental states (*e.g.* how happy did you feel today?), negative mental states (*e.g.* how anxious did you feel today?), event appraisal (*e.g.* how stressful was the most stressful event that happened today?), and substance use (*e.g.* how much soft drugs did you use today?). These items were rated on a visual analogue scale ranging from 0 (not at all) to 100 (very much). A list of all diary items has been reported elsewhere [40].

### Diagnostic interview

Immediately before and after the diary procedure, the short version of the Schedules for Clinical Assessment in Neuropsychiatry (mini-SCAN) was administered by trained researchers (MJS, RNG and a research assistant supervised by MJS and RNG) [44]. The mini-SCAN is a semi-structured diagnostic interview that assesses whether individuals meet the criteria for mental disorders, including mood, anxiety, psychotic, and substance use disorders, and attention-deficit hyperactivity disorder. Further, the mini-SCAN includes a screener for autism spectrum disorder. The mini-SCAN was complemented by the aggressive behavior subscale of the Adult Self Report [45] (ASR) to also include oppositional or antisocial behavior. The mini-SCAN and ASR were administered by reading out the questions and rating participants’ answers by trained interviewers. The interview assessed the severity of symptoms in the past month (at baseline, before the diary period) and in the past six months (at post, after the diary period). Symptoms were scored as either absent (coded 0), sub-threshold (coded 1), or clinical (coded 2) [40]. The sum score of all items was considered to reflect the severity of global symptoms and could range from 0 to 450.

### Analysis

First, we selected diary items that reflected mood and were balanced in terms of valence (*i.e.,* an equal number items reflecting positive and negative mental states were chosen). This yielded 10 items in the first (single case) dataset (5 positive, 5 negative valence) and 28 items in the second dataset (14 positive, 14 negative valence). Examples of such items are “feeling relaxed” and “feeling down”. Lists of all items assessed in the TRAILS TRANS-ID study, the items we selected for analyses, and our motivation for selecting these items is provided in the supplement. For each individual separately, we iteratively performed principal component analyses within sliding windows (*i.e.,* segments of the time series). For the first dataset (*N*=1, length = 1478 completed observations), these windows contained 150 observations, amounting to 1326 windows in total. This window size corresponds to the size adopted by Lever and colleagues [33], who used windows containing 10% of the time series length. For the second dataset (*N*=122, length = ±183 observations per person), windows contained a maximum of 60 observations, resulting in on average 123.91 windows per person (SD=4.96). Here, window size was chosen to strike a balance between overfitting (*i.e.,* using small windows, for instance, containing 10% of the observations, which would complicate reliable principal component analyses) and underfitting (*i.e.,* using large windows and potentially smoothing over potentially meaningful trends), while taking into account that on average 11.45% of the observations was missing. We inspected the influence of this methodological choice in sensitivity analyses, where we used windows of 40 and 80 observations.

**Fig. 1:**
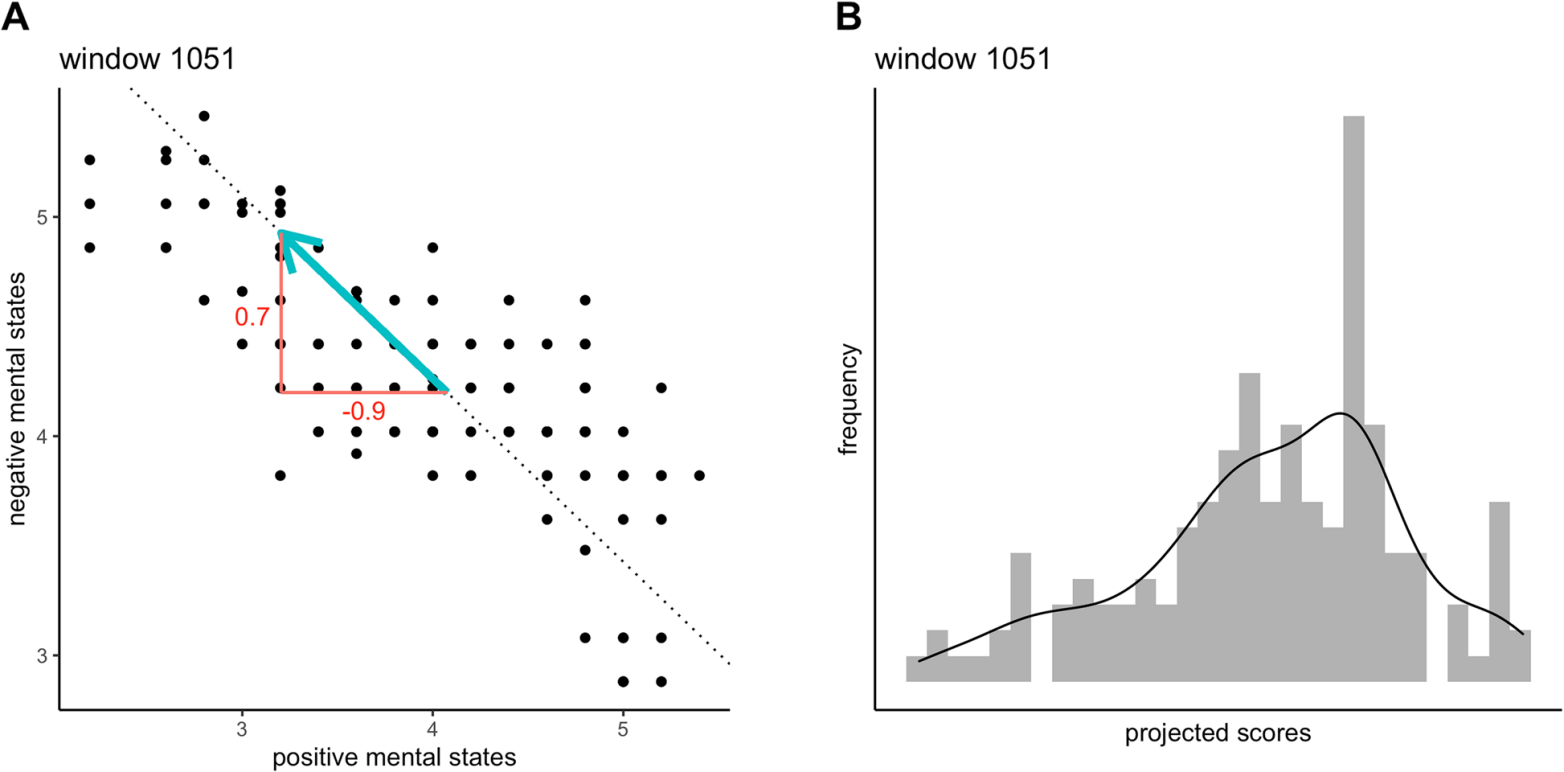
Example of a principal component analysis performed in a single window from the first dataset [39]. The left figure shows the mean positive and negative mental states of the assessment occasions in this window (indicated by black dots), as well as the first principal component (dotted line). The blue arrow reflects the vector that was inferred from the principal component analysis: the length of the arrow corresponds to the proportion of variance explained by the first principal component, while the direction of the arrow corresponds to the skewness of the scores projected on this component. The skewness, illustrated in the right plot, is directed towards the left. Together, the length and direction of the vector can be used to infer the predicted change in mental states. Here, we would predict an increase in negative mental states (0.7) and a larger decrease in positive mental states (-0.9). A similar, more detailed explanation of this method was described by Lever and colleagues [33]

Within each window, we retrieved (i) the amount of variance explained by the first principal component and (ii) the skewness of the scores projected on this first principal component. Together, these parameters formed a vector in a two-dimensional space (Fig. 1). The length of this vector corresponds to the variance explained by the first principal component (*i.e.,* the largest eigenvalue of the covariance matrix). We considered this length reflective of the structure of mental states: it captures to what extent mental states are interrelated and unidimensional (Fig. 1). In line with earlier studies, we expected the vector to lengthen prior to symptom transitions, meaning that each window should have a larger vector than the previous windows [33, 34, 46, 47]. Provided that there is no change in overall variance, this corresponds to an increased symptom covariance (as previously observed in group-level studies [17–24, 48–50]). From the length and direction of the vector, we inferred the expected change in positive and negative mental states (Fig. 1). Repeating this procedure for each window resulted in a time series that described the expected change in mental states over time for a particular person. The trend in these time series was computed using Kendall’s tau correlation coefficient. Kendall’s tau is a nonparametric correlation coefficient that assesses the similarity in the rank order of two variables, without making assumptions about the linearity of the trend [51]. A similar approach has been described before by Lever and colleagues [33].

The first dataset was analyzed to provide a proof of concept. Here, we examined whether the known relapse in depression (which occurred around day 127) was preceded by a corresponding predicted increase in negative mental states. We expected that the predicted increase in negative mental states would be small long before the transition, and would rise as the transition approached. This was quantified using Kendall’s tau, which is referred to here as the indicator. A significant, positive tau (*P*<.05) was considered indicative of a meaningful trend in the predicted change in mental states.

The second dataset was analyzed to investigate whether the aforementioned approach also holds for smaller symptom changes as experienced by at-risk individuals. Here, we analyzed whether the difference in symptom severity before and after the diary period corresponded to the predicted change in daily mental states. We focused on predicted changes in *negative* (and not positive) mental states because these changes conceptually matched our outcome (in- or decreases in symptom severity). We expected that large reductions in symptoms would coincide with a large predicted decrease in negative mental states, and vice versa. Again, trends in predicted changes in mental states were inferred from Kendall’s tau. The accuracy of the indicator was computed as the percentage of individuals for whom a change in symptoms (increase or decrease) was preceded by a corresponding trend in the predicted change in negative mental states. Compared to the change in symptoms in the first data set, the change in symptoms in the second study was smaller and not always of similar clinical significance. Therefore, we examined to what extent the accuracy of the indicator was dependent on the magnitude of symptom shifts or the clinical status of participants (*i.e.,* with vs. without diagnosis).

Analyses of the second dataset differed from those of the first dataset in three ways. First, the iterative principal component analyses were done on sum scores of positive and negative mental states rather than individual items. This was done because in some of the windows, items loaded ambiguously on the first principal component, which complicated deriving the orientation of this component in a two-dimensional space (Fig. 1). Second, in the second study, the skewness of the projected scores was sometimes close to 0. This caused 180-degree shifts in the predicted direction across multiple consecutive windows, as also reported by Lever and colleagues [33]. We corrected this by reversing “deviant” directions: when the direction of the indicator in windows 1 to 5 was positive, positive, negative, positive, positive – meaning that the first and last two windows suggested an increase in positive mental states, while the third window suggested an increase in negative mental states – we corrected the third window by reversing the predicted change scores. This is equivalent to 'flipping' the vector in Fig. 1. In the supplement, we also report the results obtained with a different skewness correction, namely, removing the directions that were based on nonsignificant skews. A third and final difference concerns the significance test of the indicator. Specifically, we evaluated the overall accuracy of the indicator in the second study through permutation testing, which involved shuffling the time order of each individual’s data and then computing the indicator. In this shuffled dataset, the temporal ordering of the data was lost, and therefore, we would expect the indicator to perform worse compared to the original data. Permutations were repeated 200 times to obtain a stable accuracy estimate. This estimate was then compared to the accuracy based on the original (non-shuffled) data [52]. Because accuracy was computed across individuals, we could not take a similar approach in the first study. Analyses were performed using R (version 4.0.2) [53].

## Results

### Study 1

**Fig. 2:**
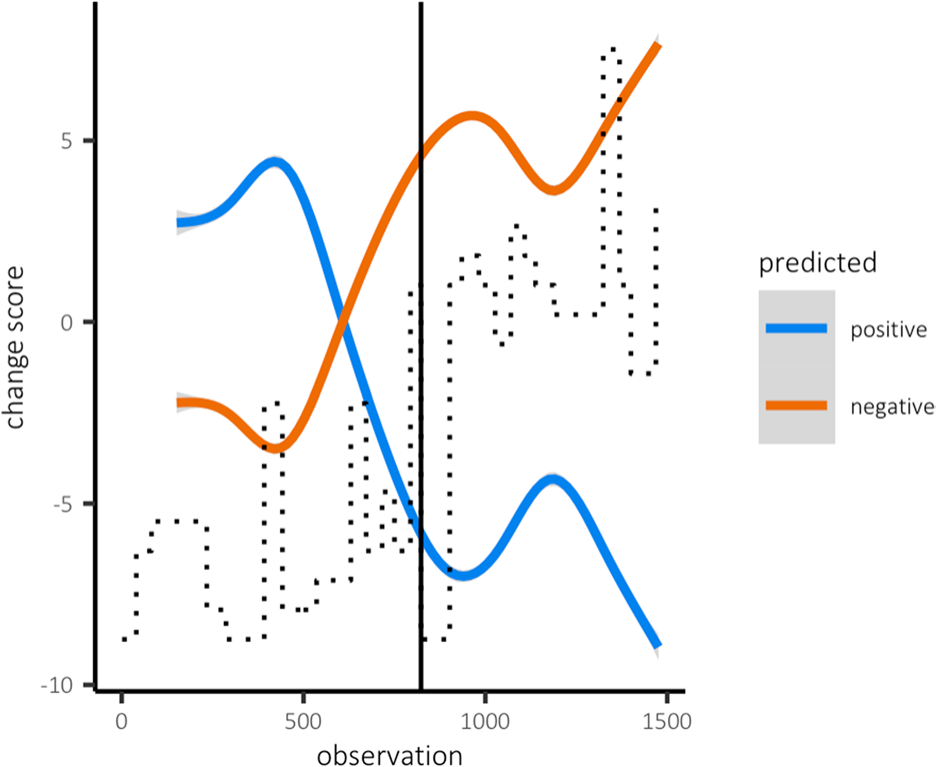
The predicted change in positive (red) and negative (blue) mental states. The dotted line depicts the weekly assessed severity of depressive symptoms based on the SCL-90. The black line marks the relapse in depressive symptoms, which occurred around day 127 (observation 823)

On average, the participant completed 6.2 (SD = 1.9) assessments per day, amounting to 1,478 diary entries in total [39]. Prior to the relapse in depression, around day 127, there was a rising trend in the predicted change in negative mental states (tau = 0.68, *P*<.01; Fig. 2). First, from day 54 to day 88 (observations 384-614) the predicted *reduction* in negative mental states became *smaller*. From day 89 (observation 615) onward, the indicator predicted an accumulating increase in negative mental states together with a decrease in positive mental states. Therefore, starting more than 2 months prior to relapse, a rising trend in the predicted change in negative mental states appeared to 'warn' for the transition. This trend did not end immediately after the transition, supporting the idea that there might be a continuous relation between the trend in predicted changes in mental states and impending symptom change.

### Study 2

Sample characteristics are provided in Table 1. Individuals who completed the diary period, as well as both diagnostic interviews (*N*=122) filled in on average 162 diary entries (88.55%, SD = 17.51, range = 114-189). During the month *before* the diary study, 40 individuals (32.79% of the total sample) met the criteria for at least one psychiatric disorder. *During* the diary period, 34 individuals (27.87%) met the criteria for at least one psychiatric disorder (Table 1). The sum of the items that were rated in the diagnostic interviews ranged from 3-241 (baseline) and 2-230 (post), with average sum scores of 66.25 (baseline; SD = 42.71) and 69.78 (post; SD = 46.89). Based on the diagnostic interview data, approximately half of the sample improved in terms of their symptoms (*N*=60, 49.18%), while the other half reported worse symptoms at post compared to baseline (*N*=58, 47.54%) or had an equal symptom severity at baseline and post (*N*=4, 3.28%). The absolute magnitude of symptom transitions varied between 1, which has no clinical significance, and 108, which signifies an in- or decrease in the severity of half of the items assessed in the diagnostic interview (mean change=18.46, SD=18.73, median=12.00). For the majority of individuals (*N*=93, 76.23%), symptom transitions did not lead to a change in diagnosis. For others (*N*=29, 23.77%), symptom transitions coincided with a change in diagnostic status: 11 individuals no longer met criteria for (one of) their diagnosis and 18 met criteria for a new diagnosis.

**Table 1:** Sample characteristics

	Baseline (pre-diary)	Post (post-diary)
	*N*=134	*N* = 122
Sex (% males)	76 (56.7%)	69 (56.6%)
Age (SD)	23.6 (0.7)	23.6 (0.7)
No. of completed diary entries		162 (88.6%)
*mini-SCAN (N, %)*		
Anxiety disorder	21 (16%)	12 (10%)
Mood disorder	28 (21%)	23 (19%)
Psychotic disorder	2 (1%)	5 (4%)
Attention deficit and/or hyperactivity disorder	8 (6%)	8 (7%)
Substance use disorder	3 (2%)	4 (3%)
Autism spectrum disorder^a^	32 (24%)	31 (25%)
Aggressive behavior subscale ASR	5.3 (4.4)	5.5 (4.8)
Sum score (SD)	66.3 (42.7)	69.8 (46.9)

^a^Note that this reflects a positive score on a screener, rather than a diagnosis

*ASR* Adult Self Report, *Mini-SCAN* short version of the Schedules for Clinical Assessment in Neuropsychiatry

**Fig. 3:**
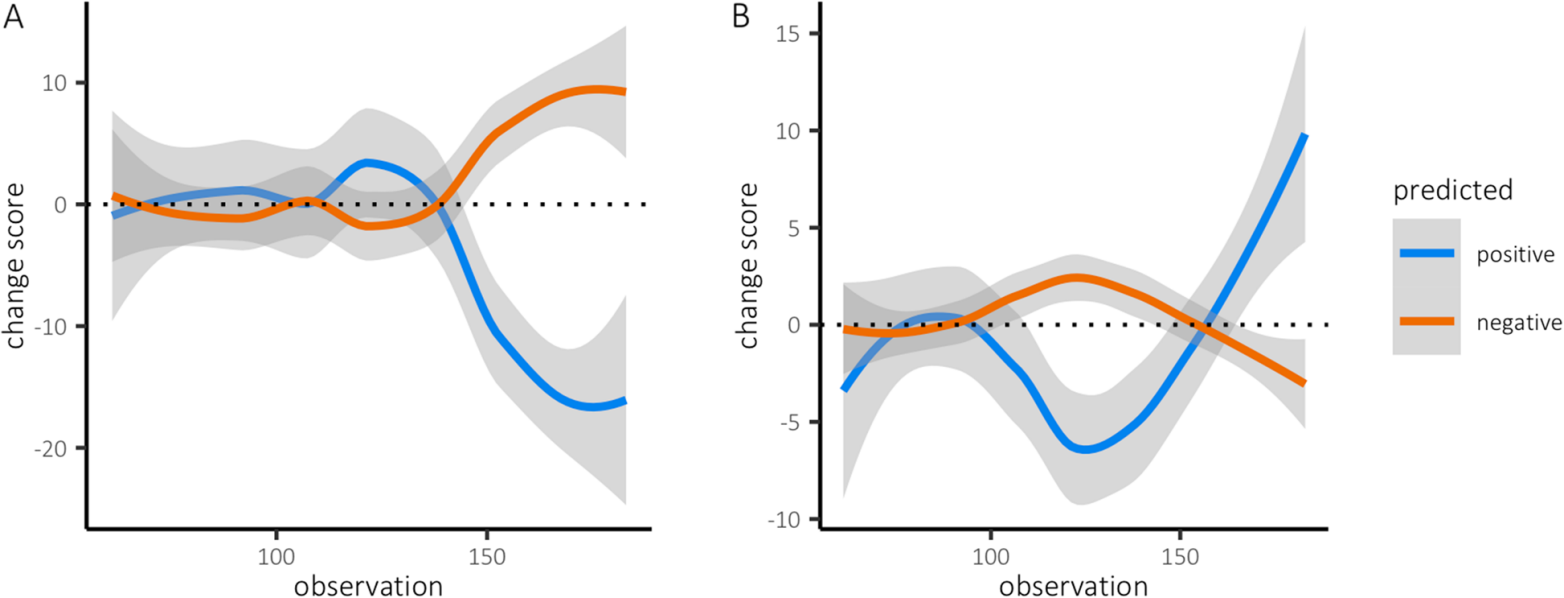
Illustration of the predicted change in positive and negative mental states for two individuals. Because windows spanned 60 observations, the earliest prediction was made for the 60^th^ observation. **A** This individual reported an increase in symptom severity based on the sum score of the diagnostic interview (baseline=73; post=96). This symptom increase is preceded by a gradual rise in the predicted change of negative mental states (tau=0.25, *P*<0.05). **B** This individual reported a reduction in symptom severity (baseline: 63, post: 41), which was preceded by a gradual decline in the predicted change in negative mental states (tau=-0.29, *P*<0.01)

For 65 individuals (53.28%), we found a significant change in their predicted change in negative mental states (mean absolute tau=0.18, SD=0.13, range=0-0.56). Specifically, in 35 individuals (28.69%) we found a rising trend in the predicted change in negative mental states, which would suggest future symptom worsening (mean tau=0.28, SD=0.13, range=0.14-0.56). The other 30 individuals (24.59%) showed a declining trend in their predicted change in negative mental states, implying improvement in symptoms (mean tau=-0.27, SD=0.10, range=-0.45 to -0.14). Examples of both trends are depicted in Fig. 3.

**Fig. 4:**
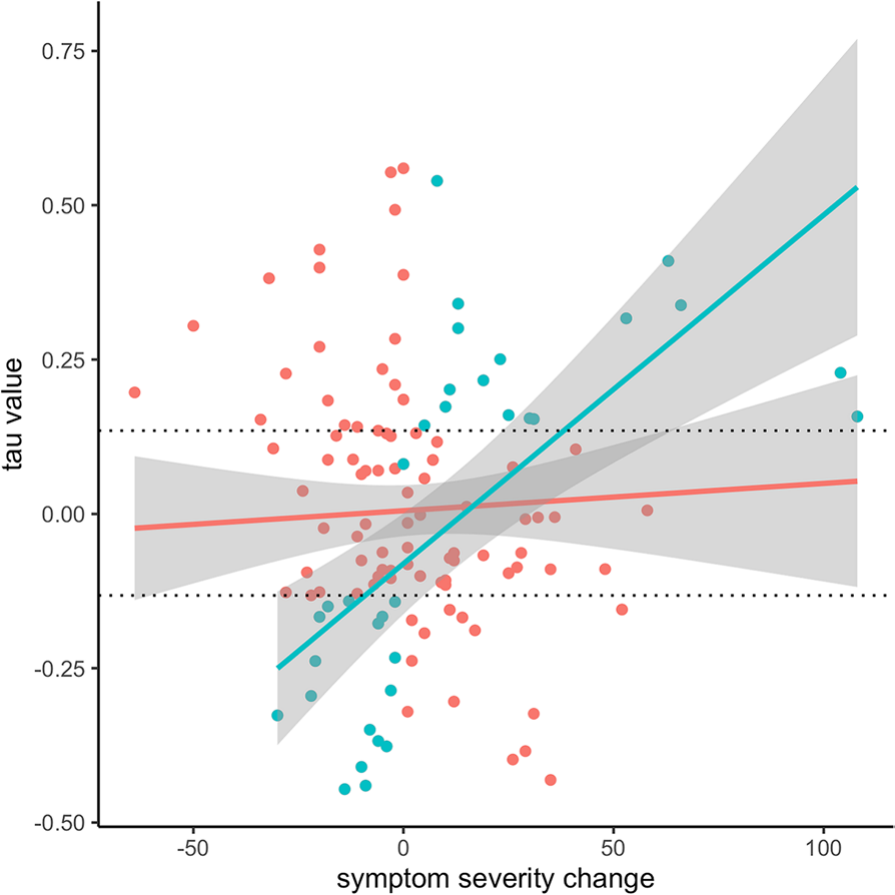
The association between the trend in the predicted change in negative mental states (tau) and change in symptom severity from baseline to post in individuals for whom the indicator was correct (*N*=34, blue) and in the entire sample (*N*=122, red). Horizontal dotted lines depict the threshold for significant (*P*<.05) versus non-significant (P>.05) values of tau. For all individuals (dots) above and below these dotted lines (*N*=65), a change in symptoms was predicted. Negative symptom change implies improvement of psychopathology (i.e., a reduction of symptoms over time). For individuals for whom such improvement was predicted by the indicator, *larger* reductions in symptoms were related to more pronounced trends in the indicator (*i.e.* more negative values of tau). Vice versa, individuals for whom symptom worsening was predicted correctly showed a more pronounced indicator (i.e., more positive values of tau) as symptom change increased

The trend in the predicted change of negative mental states correctly predicted symptom changes in 34 individuals (27.87% of the entire sample), yielding a true positive rate of 52.31% (34/65). Of these individuals, 16 reported more severe symptoms at post, 17 reported less severe symptoms at post, and 1 did not change in terms of symptom severity. For this latter individual, the correct prediction reflected a nonsignificant trend in tau. For the others, correct predictions were reflected by significant up- or downward trends in tau, which became more pronounced as the absolute change in symptom severity increased. This was not the case for the entire sample (*i.e.,* including those individuals for whom the indicator was not predictive of symptom change; Fig. 4). Individuals for whom the indicator worked did not differ from others in terms of their absolute change in symptoms (mean absolute symptom change 22.79 vs. 15.94, Cohen’s d=1.33, Welch’s t(40.69)=1.43, *P*=0.16). Further, the accuracy of the indicator was not related to the likelihood of meeting the diagnostic criteria at the beginning of the study (2 (1) = 0.01, *P* =.93) or after (2 (1) = 0.21, *P* =.64). Nevertheless, the performance of the indicator improved when evaluated in subsets of individuals with large symptom changes in either direction. Specifically, in the 0.50 quantile (*i.e.,* individuals whose absolute symptom change exceeded the median absolute symptom change), the accuracy of the indicator was 32.76%. In the 0.25 and 0.10 quantiles, accuracy equaled 26.67% and 41.67%. By definition, these latter samples are relatively small – comprising 25% and 10% of individuals (i.e., *N*=30 and 12) – and hence, these results warrant cautious interpretation.

In shuffled data, where the temporal structure of the data was lost, the indicator reached an accuracy of 20.63%. This accuracy was not significantly different from the accuracy obtained in the original data (27.87%, z=-1.32, *P*=.09), and hence, we could not rule out the possibility that the indicator’s accuracy was due to chance rather than critical slowing down. However, in sensitivity analyses where we only retained predictions that were based on significantly skewed projected scores, the indicator performed significantly better (accuracy 22.92%) compared to permutation tests (accuracy 11.76%, z=-2.04, *P*=.02; supplement). The accuracy of the indicator did not significantly change when considering alternative window sizes (window of 40 observations: accuracy 19.67%, z=1.50, *P*=.07; window of 60 observations: accuracy 24.59%, z=0.58, *P*=.28; see supplement for further details).

## Discussion

We investigated whether symptom changes within individuals – involving either an increase or decrease in symptoms over time – can be predicted based on the direction of critical slowing down in daily reports of mental states [33]. First, we used data from a middle-aged man with a history of major depression who monitored his mood ten times per day for almost eight months. After four months, he experienced a relapse of depression, which was preceded by critical slowing down [8]. Building on this previous work, we found that critical slowing down not only anticipated the symptom transition, but also signified that the transition was directed toward an increase (as opposed to a decrease) in symptoms. We next investigated whether the same holds on a larger scale, for more diverse symptom transitions (*i.e.,* increases and decreases of varying magnitude). To this end, we analyzed data from 122 young adults at increased risk for psychopathology who monitored their mental states daily for six consecutive months. For one in four (main analysis) to five (sensitivity analysis) individuals, the directionality of critical slowing down correctly predicted their reported symptom change. However, the indicator’s accuracy only exceeded chance levels in sensitivity analysis, and therefore, the support for directionality of critical slowing down as a predictor of future symptom changes was less robust in study 2 than in study 1. In conclusion, results from study 1 and 2 tentatively support the idea that for *some* individuals, particularly those with large symptom changes, the direction of critical slowing down matches the direction of future symptom transitions.

Our findings tentatively suggest that anticipating the direction of symptom transitions by means of critical slowing down may be limited to specific individuals. We might get a better idea of who these individuals might be by taking a closer look at the literature that described parameters that determine whether the direction of critical slowing down can be used to infer the future state of a system. First, critical transitions in dynamical systems can be the result of either a positive feedback loop, or a negative feedback loop with delayed effects [54]. The direction of critical slowing down most accurately predicts the future if the system’s dynamics are governed by positive feedback loops [33]. In the context of psychopathology, this means that mental states should amplify each other: *e.g.,* when feeling tired leads to feeling down, which leads to concentration problems, which leads to feeling more tired. From a complex dynamic systems perspective, such feedback loops give rise to self-sustaining states (*e.g.,* a mental disorder). Such self-sustaining states, and the feedback loops that underlie them, have been linked to both past and present mental disorders. That is, both individuals with a past mental disorder and individuals with a current mental disorder have been shown to have relatively densely connected symptoms, albeit in different studies. For example, network studies [20–24, 48–50] showed that symptoms are more densely connected in remitted individuals compared to treatment-seeking individuals with mental health problems. Provided that these group-level findings generalize to the level of the individual, this could mean that feedback loops strengthen as symptoms decline in severity. At the same time, however, cross-sectional network studies [55–57] and dynamic network studies [58, 59] showed that individuals with a mental disorder have higher connectivity than non-affected individuals – suggesting that those with a disorder, too, have strong feedback loops^1^. These seemingly discrepant findings could be reconciled if strengthened feedback loops between mental states reflect a scar imposed by mental disorders [60]. This would mean that feedback loops strengthen with illness duration – which received tentative support [61, 62]. This has consequences for the utility of the indicator presently studied, which depends on the strength of feedback loops. Specifically, it would mean that the indicator might be more suitable for individuals with a longer illness duration. In agreement with this, we found that the indicator clearly matched the symptom course in study 1, which concerned a remitted individual with a history of major depression that dated back 30 years [63], while it was less robust in study 2, which concerned individuals with a shorter illness duration. Besides illness duration – and by analogy, the strength of feedback loops – the participant in study 1 differed from the participants in study 2 in the magnitude of symptom transitions. Specifically, participants in the latter study generally reported smaller, perhaps more gradual, symptom transitions compared to the participant in study 1. This touches upon a second factor that determines the accuracy of the indicator in exposing the direction of critical slowing down. That is, the indicator is more accurate when transitions reflect full collapses, as opposed to (sequential) partial collapses [33]. This could mean that a transition in a small set of specific symptoms (*i.e.,* a partial collapse) is more difficult to detect than a change in almost all symptoms (*i.e.,* a full collapse). In line with this, present findings tentatively suggest that the direction of larger symptom transitions (as observed in study 1, and subsets of individuals from study 2) is more predictable than that of smaller symptom transitions. In conclusion, it is possible that critical slowing down and its direction are only detectable in individuals with a long illness duration (implying strong feedback loops) who experience relatively large transitions.

### Critical slowing down: sudden versus gradual transitions

Critical slowing down is often considered prior to sudden transitions, such as the collapse of an ecosystem [64, 65]. A relapse in depression, as examined in study 1, might be of comparable impact and suddenness [8]. Critical slowing down anticipates such transitions if they occur through a cusp (or saddle node) bifurcation. This assumption can indirectly be verified, for instance by testing for bimodality and hysteresis [29]. A recent study confirmed that the data we analyzed in study 1 indeed shows such “signs of complexity” [66]. Hence, the data analyzed in study 1 likely meet the requirements to observe critical slowing down. For the data analyzed in study 2, we could not verify whether symptom shifts indeed occurred through the specific types of bifurcations related to critical slowing down. Yet, this applies to the majority of applied studies into critical slowing down [8, 31, 67]. For these studies, it is uncertain whether critical slowing down should be expected at all. This makes it difficult to assess whether (not) observing critical slowing down reflects a true or false positive (or negative). We addressed this ambiguity by shuffling the temporal order of the data analyzed in study 2. If critical slowing down and its direction would still be detected in such shuffled data, it likely reflects a false positive. We found that this was not the case, provided that the direction of critical slowing down is inferred using conservative criteria (sensitivity analyses). Still, it remains uncertain whether the shifts that occurred in Study 2 resembled the type of shifts for which critical slowing down and its direction are informative. It thus requires further research to translate the mathematical assumptions of critical slowing down to empirical settings.

### Strengths and limitations

A first strength of the current study is its ability to inspect critical slowing down *within individuals*. In contrast to group-level studies, we could therefore directly test the hypothesis that the direction of critical slowing down is informative of impeding symptom transitions. Second, we used a variance-based method for detecting the direction of critical slowing down, which is less sensitive to the timescale of assessments compared to other metrics of critical slowing down such as the autocorrelation [68]. Additionally, variance-based methods are less dependent on the amount of available data for each individual [33]. It remains possible, however, that differences in the sampling frequency and duration (study 1: 1478 observations for one participant; study 2: max. 183 observations for 122 participants) contributed to differences in power, perhaps explaining why the indicator was more robust in study 1 compared to study 2. At the same time, critical slowing down only has practical relevance if it can be detected in data that are feasible to collect. Hence, although a relatively short sampling duration might have compromised statistical power in study 2, the data quantity in this study likely comes closer to what would be seen in applied settings (*e.g.,* clinical practice) compared to the data quantity in study 1.

Several limitations should be taken into account when considering the present findings. First, there were no measures of symptom severity *during* the diary period in study 2, and therefore, we cannot rule out the possibility of unnoticed sudden symptom transitions during the diary period. For instance, it is possible that the symptom changes experienced by the at-risk individuals in study 2 did not evolve gradually, but rather, occurred suddenly during the diary period (*e.g.,* after 4 months). This, in turn, should affect the timing of critical slowing down: gradual development implies critical slowing down towards the end of the diary period [69–72], while sudden development during the diary period implies an earlier manifestation of critical slowing down (*e.g.,* between 2-4 months). Since we inspected critical slowing down across the diary period (toward the end), overlooking some symptom transitions may have led to an underestimated accuracy of the indicator. A second limitation is that we inspected only two directions of critical slowing down (towards an increase vs. decrease in symptoms). This might be an oversimplification, as more nuanced directions could also be considered (*e.g.,* towards an increase in depression vs. anxiety vs. aggression) [73]. At the same time, inspecting such fine-grained directions seems only warranted if the indicator would correctly distinguish between more global directions. Further, critical slowing down is less pronounced in high-dimensional systems compared to lower dimensional systems [35, 71], meaning that considering many potential directions might lower the detectability of our indicator and would require a larger amount of data. Hence, reducing the complexity of our data seemed desirable.

## Conclusion

In ecology, critical slowing down not only anticipates symptom transitions but also informs on the direction of these transitions [33]. If the same holds for psychopathology, we could detect whether a specific individual, at a specific moment in time, is increasingly likely to experience a specific type of symptom transition (in this case, towards either improvement or worsening). Based on two studies, we found tentative support for the idea that the direction of critical slowing down might anticipate the direction of impeding transitions, provided that these transitions are sufficiently large. Inconclusive findings preclude firm conclusions, and more research into critical slowing down in the context of symptom transitions is necessary. A promising step forward could be to empirically investigate to what extent critical slowing down depends on the strength of feedback loops. One way of addressing this could be to compare critical slowing down between individuals with strongly versus weakly connected symptom networks. Second, future studies are necessary in order to explicitly address the assumptions underlying critical slowing down (*e.g.,* with respect to the bifurcation that describes the transition). This requires a translation from mathematical theory [74] to empirical reality, which could perhaps be facilitated by formal modeling [75]. Ultimately, such a translation will improve our ability to identify those individuals whose system of mental states matches the behavior of other complex dynamic systems. These are the individuals who may benefit from the anticipatory capacity of critical slowing down. A clearer picture of when and for whom complex dynamic systems principles apply will crucially determine their clinical utility.

## Supplementary Information

**Additional file 1.** Description of data: detailed explanation of item selection and two sensitivity analyses (one with an alternative skewness correction, one with alternative window sizes)

## Data Availability

The dataset of the first study is publicly available in the Open Psychology Data repository (https://openpsychologydata.metajnl.com/articles/10.5334/jopd.29/). The dataset of the second study is not publicly available due to the possibility to identify participants based on their daily reports and clinical data (European law).

## Abbreviations

ADHD: Attention deficit hyperactivity disorder
SCL-90: Symptom checklist 90
SD: Standard deviation

## Footnotes

1 Note that both longitudinal and cross-sectional network studies should be cautiously interpreted, since comparing network characteristics between individuals with differing levels of symptom severity might be subject to Berkson’s bias or collider bias [9].
