# Supplementary material for: Anticipating the direction of symptom progression using critical slowing down: a proof-of-concept study
Source: BMC Psychiatry. 2022 Jan 21;22:49. doi: 10.1186/s12888-022-03686-9 (PMC8781362; doi:10.1186/s12888-022-03686-9)
Supplement: Supplementary file 1 — Additional file 1. Description of data: detailed explanation of item selection and two sensitivity analyses (one with an alternative skewness correction, one with alternative window sizes) [file 12888_2022_3686_MOESM1_ESM.docx]

**Supplement**

Anticipating the direction of symptom progression using critical slowing down: a proof-of-concept study

Marieke J. Schreuder, Johanna T.W. Wigman, Robin N. Groen, Els Weinans^2^, Marieke Wichers, Catharina A. Hartman

1. Items

For study 1, the experience sampling questionnaire contained 12 mood-related items. In contrast to study 2, where items assessed individuals’ mood during the past day retrospectively, the items in study 1 assessed present mood (*i.e.* at the moment of filling in the questionnaire; table S1). To balance the number of positive and negative mental states, we left out two randomly selected negative mood items from analyses (I feel indecisive; I feel suspicious). This resulted in five positive and five negative mental states that were further analyzed (table S1).

For study 2, the daily diary assessments consisted of 58 items (Table S1), of which 52 pertained to mood (17 positive mental states, 35 negative mental states). Since items do not necessarily have the same meaning between individuals, aggregating all items would likely result in noisy variables. We therefore decided to select those items that were most likely to reflect the same two constructs (mental health vs. ill-health) across individuals. To this end, we performed a principal component analysis, accounting for the variance explained by individuals and the temporal ordering of the data. Briefly, we fitted multilevel models where each of the 52 mood-related items was predicted by an intercept and time. Both were allowed to vary between individuals. Further, we specified that observations from the same individual were autocorrelated. These autocorrelations were again allowed to vary between individuals. The residuals of these models reflected the variability in observations that was not due to either individual differences, time trends or autocorrelation. Using these residuals, we fitted a principal component analysis with two components. These two components would eventually reflect the directions in which individuals could change (*i.e.,* towards mental health/positive mental states vs. towards mental ill-health/negative mental states). We inspected the loadings of items on the two components, and selected those positive mental states with a loading >.80 for analysis. This yielded 14 positive mental states, which were then matched to the 14 negative mental states with the highest loadings (table S2).

Table S1. Items assessed in the TRAILS TRANS-ID study

| Item |
| --- |
| 1. Last night I slept well |
| 1. I felt energetic |
| 1. I was physically active |
| 1. I was happy |
| 1. I could enjoy things |
| 1. I felt good |
| 1. I felt relaxed |
| 1. My day was worthwhile |
| 1. I could handle the things that confronted me |
| 1. Everything came easy |
| 1. I felt like undertaking things |
| 1. I am looking forward to tomorrow |
| 1. My concentration was good |
| 1. I felt appreciated |
| 1. I felt like others liked me |
| 1. I felt at ease with others |
| 1. I was social |
| 1. I felt lonely |
| 1. I felt empty |
| 1. I felt down |
| 1. I felt guilty |
| 1. I could not bring myself to do anything |
| 1. I felt tired |
| 1. I avoided things |
| 1. I was afraid of making mistakes |
| 1. I had difficulty making decisions |
| 1. I worried |
| 1. My thoughts would not leave me alone |
| 1. My mind was wandering |
| 1. I felt restless |
| 1. I felt nervous |
| 1. I was jumpy |
| 1. I felt stressed |
| 1. I felt overwhelmed |
| 1. I was easily upset |
| 1. I felt overstimulated |
| 1. It bothered me that things did not go as expected |
| 1. I was bored quickly |
| 1. I felt impatient |
| 1. I was unable to sit still |
| 1. I was impulsive |
| 1. I felt irritated |
| 1. Others felt annoyed by me |
| 1. I had a fight |
| 1. I felt rebellious |
| 1. Things did not bother me |
| 1. I had moodswings |
| 1. I felt strange |
| 1. I felt suspicious |
| 1. I experienced physical pain |
| 1. I experienced physical discomfort |
| 1. I drank alcohol today |
| 1. I used (soft)drugs today |
| 1. Number of pleasant events |
| 1. Intensity of pleasant events |
| 1. Number of unpleasant events |
| 1. Intensity of unpleasant events |
| 1. I’m dreading something that will happen soon |

Table S2. Items included in analyses

|  | Positive mental states | Negative mental states |
| --- | --- | --- |
| Study 1 (case study) | 1. I feel relaxed 2. I feel satisfied 3. I feel enthusiastic 4. I feel cheerful 5. I feel strong | 1. I feel down 2. I feel anxious 3. I feel irritated 4. I feel lonely 5. I feel guilty |
| Study 2 (TRAILS TRANS-ID study) | 1. I could enjoy things 2. I felt energetic 3. I felt at ease with others 4. I was happy 5. My day was worth living 6. I felt good 7. I felt like others liked me 8. I felt relaxed 9. I felt appreciated 10. I am looking forward to tomorrow 11. I felt like undertaking things 12. Everything came easy 13. I was social 14. Last night I slept well | 1. I felt irritated 2. I was easily upset 3. I had difficulty making decisions 4. I felt rebellious 5. I felt strange 6. I felt impatient 7. I was afraid of making mistakes 8. I was easily distracted 9. Others felt annoyed by me 10. I felt guilty 11. I had moodswings 12. I felt suspicious 13. I could not bring myself to do anything 14. I felt restless |

1. Alternative skewness correction

As described in the analysis section of the main text, the skewness of scores projected on the first principal component was sometimes close to zero. This caused 180-degree flips in the direction of the vector we calculated within consecutive windows (see Fig. 1, main text). In our main results, we corrected such flips by iteratively searching for and correcting flipping patterns (*e.g.* correcting positive – positive – negative – positive – positive to positive – positive – positive – positive – positive). Here, we report the results obtained when deleting predictions based on vectors of which the skew was not statistically significant.

We could calculate trends in the predicted change of mental states for 96 individuals (78.69%). For others, there were too many missing predictions to calculate the tau correlation coefficient across predictions. For 37 individuals (38.54 %), a significant change in their predicted change in negative mental states was found (absolute tau range = 0-0.88, mean=0.19, SD=0.18). The predicted change in negative mental states showed an increasing trend for 21 individuals (21.88%), and a decreasing trend for 17 individuals (17.71%). The trend in the predicted change of negative mental states correctly predicted symptom changes in 22 individuals (22.92 %), of whom 12 reported more severe symptoms at post, 9 reported less severe symptoms at post, and 1 did not change in terms of symptom severity. Individuals for whom the indicator worked did not differ from others in terms of their absolute change in symptoms (mean absolute symptom change 20.82 vs. 14.43, Cohen’s d=1.31, t(94)=1.57, P=0.12). Further, the accuracy of the indicator was not related to the likelihood to meet diagnostic criteria at baseline (χ^2^(1)=0.24, P=.63) or at post (χ^2^(1)<0.01, P>.99). Similar to the results reported in the main text, indicator performance improved when evaluated in subsets of individuals with large symptom changes in either direction. Specifically, in the 0.50 quantile (i.e., individuals whose absolute symptom change exceeded the median absolute symptom change across individuals), the accuracy of the indicator was 29.27%. In the 0.25 and 0.10 quantiles, accuracy equaled 25.00% and 50.00%. In contrast to our main analyses, the accuracy of the indicator significantly exceeded the accuracy obtained in shuffled data, which was 11.76% (z=-2.04, P=.02). This should however be cautiously interpreted, given that we could only inspect accuracy in the subset of individuals for whom predictions could be made (N=96).

1. Alternative window sizes

For the second study, we used window sizes of 60 observations. To determine the influence of this choice on our results, we re-ran analyses with windows containing a maximum of 40 and 80 observations. The results were largely in line with what we reported in the main text. Below, the results we obtained with window sizes 40 and 80 are denoted consecutively, with subscripts where possible. A significant change in the predicted change in negative mental states was found for 56 (45.90%, absolute tau range_40_ = 0-0.46, mean_40_=0.14, SD_40_=0.11) and 66 individuals (54.10%, absolute tau range_80_ = 0-0.64, mean_80_=0.18, SD_80_=0.14). The predicted change in negative mental states showed an increasing trend for 26 (21.31%) and 31 (25.41%) individuals. For the remaining 30 (24.59%) and 35 (28.67%) individuals, the trend was decreasing. The trend in the predicted change of negative mental states correctly predicted symptom changes in 24 and 30 individuals (accuracy_40_ = 19.67%, accuracy_80_ = 24.59%; table S3). In conclusion, the accuracy of the indicator did not seem to depend on window size.

Table S3. Results of study 2 for different window sizes

|  | Window size 40 | Window size 60 | Window size 80 |
| --- | --- | --- | --- |
| Mean number of observations per window (SD) | 36.00 (4.43) | 53.44 (6.79) | 70.85 (9.20) |
| Range of observations per window | 21.67-40.00 | 31.36-60.00 | 40.78-80.00 |
| Accuracy | 19.67% | 27.87% | 24.59% |

*Note.* The mean number of observations reflect the number of observations per window, averaged across windows (within individuals), averaged across individuals.
